# Assessment of risk of bias in translational science

**DOI:** 10.1186/1479-5876-11-184

**Published:** 2013-08-08

**Authors:** Andre Barkhordarian, Peter Pellionisz, Mona Dousti, Vivian Lam, Lauren Gleason, Mahsa Dousti, Josemar Moura, Francesco Chiappelli

**Affiliations:** 1 Oral Biology & Medicine, School of Dentistry, UCLA, Evidence-Based Decisions Practice-Based Research Network, Los Angeles, USA; 2 Pre-medical program, UCLA, Los Angeles, USA; 3 School of Medicine, Universidade Federal de Minas Gerais, Belo Horizonte, Brazil; 4 Evidence-Based Decisions Practice-Based Research Network, UCLA School of Dentistry, CHS 63-090, 10833 Le Conte Avenue, Los Angeles, CA, 90095-1668, USA

## Abstract

Risk of bias in translational medicine may take one of three forms: A. a systematic error of methodology as it pertains to measurement or sampling (e.g., selection bias), B. a systematic defect of design that leads to estimates of experimental and control groups, and of effect sizes that substantially deviate from true values (e.g., information bias), and C. a systematic distortion of the analytical process, which results in a misrepresentation of the data with consequential errors of inference (e.g., inferential bias). Risk of bias can seriously adulterate the internal and the external validity of a clinical study, and, unless it is identified and systematically evaluated, can seriously hamper the process of comparative effectiveness and efficacy research and analysis for practice. The Cochrane Group and the Agency for Healthcare Research and Quality have independently developed instruments for assessing the meta-construct of risk of bias. The present article begins to discuss this dialectic.

## Background

As recently discussed in this journal [1], translational medicine is a rapidly evolving field. In its most recent conceptualization, it consists of two primary domains: translational research proper and translational effectiveness. This distinction arises from a cogent articulation of the fundamental construct of translational medicine in particular, and of translational health care in general.

**Figure 1:**
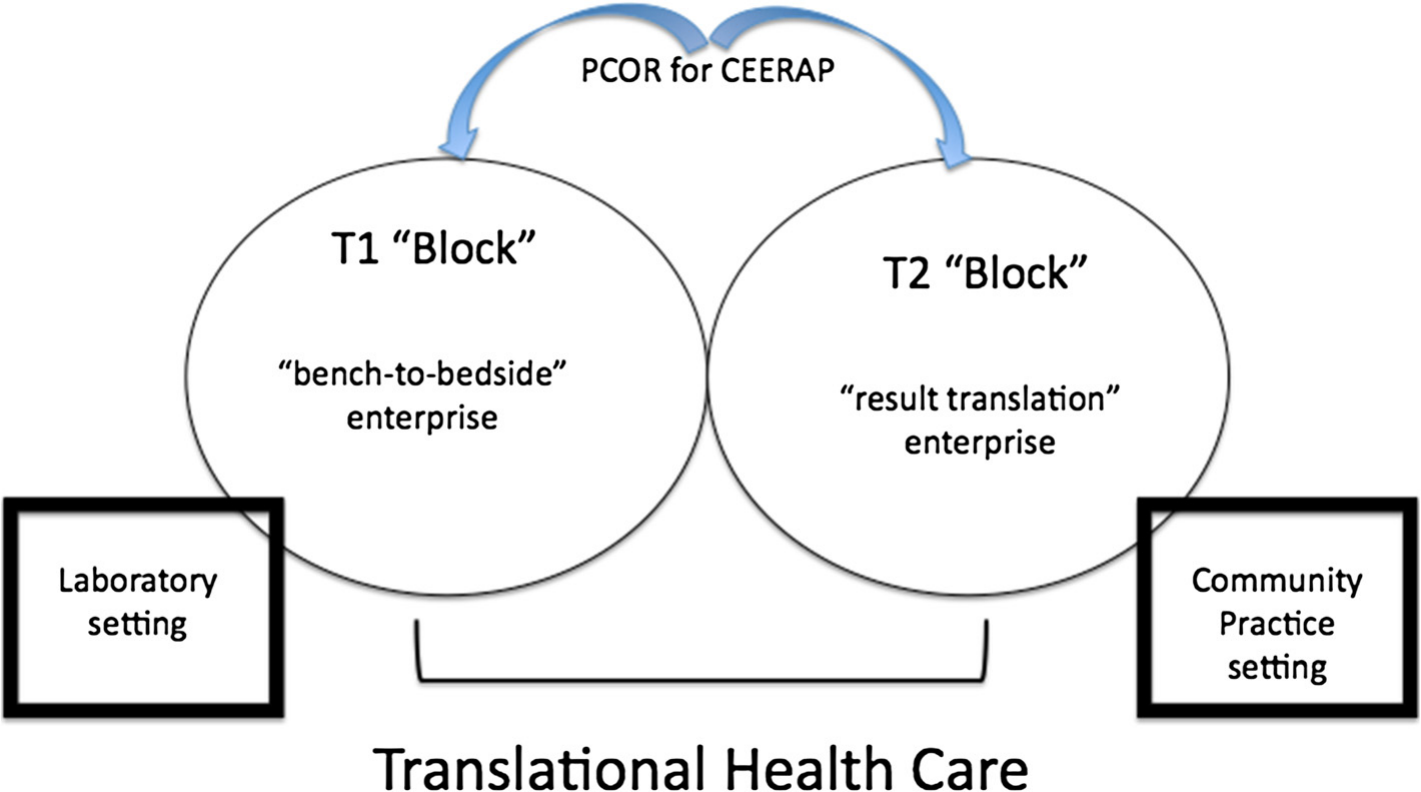
**Schematic representation of the meta-construct of translational health care in general, and translational medicine in particular, which consists of two fundamental constructs: the T1 “block” (as per Institute of Medicine's Clinical Research Roundtable nomenclature), which represents the transfer of new understandings of disease mechanisms gained in the laboratory into the development of new methods for diagnosis, therapy, and prevention as well as their first testing in humans, and the T2 “block”, which pertains to translation of results from clinical studies into everyday clinical practice and health decision making [**[3]**].** The two “blocks” are inextricably intertwined because they jointly strive toward patient-centered research outcomes (PCOR) through the process of comparative effectiveness and efficacy research/review and analysis for clinical practice (CEERAP). The domain of each construct is distinct, since the “block” T1 is set in the context of a laboratory infrastructure within a nurturing academic institution, whereas the setting of “block” T2 is typically community-based (e.g., patient-centered medical/dental home/neighborhoods [4]; “communities of practice” [5]).

The Institute of Medicine’s Clinical Research Roundtable conceptualized the field as being composed by two fundamental “blocks”: one translational “block” (T1) was defined as “…the transfer of new understandings of disease mechanisms gained in the laboratory into the development of new methods for diagnosis, therapy, and prevention and their first testing in humans…”, and the second translational “block” (T2) was described as “…the translation of results from clinical studies into everyday clinical practice and health decision making…” [2]. These are clearly two distinct facets of one meta-construct, as outlined in Figure 1. As signaled by others, “…Referring to T1 and T2 by the same name—translational research—has become a source of some confusion. The 2 spheres are alike in name only. Their goals, settings, study designs, and investigators differ…” [3].

For the last five years at least, the Federal responsibilities for “block” T1 and T2 have been clearly delineated. The National Institutes of Health (NIH) predominantly concerns itself with translational research proper - the bench-to-bedside enterprise (T1); the Agency for Healthcare Research Quality (AHRQ) focuses on the result-translation enterprise (T2). Specifically: “…the ultimate goal [of AHRQ] is research translation—that is, making sure that findings from AHRQ research are widely disseminated and ready to be used in everyday health care decision-making…” [6]. The terminology of translational effectiveness has emerged as a means of distinguishing the T2 block from T1.

Therefore, the bench-to-bedside enterprise pertains to translational research, and the result-translation enterprise describes translational effectiveness. The meta-construct of translational health care (viz., translational medicine) thus consists of these two fundamental constructs: translational research and translational effectiveness, which have distinct purposes, protocols and products, while both converging on the same goal of new and improved means of individualized patient-centered diagnostic and prognostic care.

It is important to note that the U.S. Patient Protection and Affordable Care Act (PPACA, 23 March 2010) has created an environment that facilitates the pursuit of translational health care because it emphasizes patient-centered outcomes research (PCOR). That is to say, it fosters the transaction between translational research (i.e., “block” T1) and translational effectiveness (i.e., “block” T2), and favors the establishment of communities of practice-research interaction. The latter, now recognized as practice-based research networks, incorporate three or more clinical practices in the community into a community of practices network coordinated by an academic center of research.

Practice-based research networks may be a third “block” (T3) in translational health care and they could be conceptualized as a stepping-stone, a go-between bench-to-bedside translational research and result-translation translational effectiveness [7]. Alternatively, practice-based research networks represent the practical entities where the transaction between translational research and translational effectiveness can most optimally be undertaken. It is within the context of the practice-based research network that the process of bench-to-bedside can best seamlessly proceed, and it is within the framework of the practice-based research network that the best evidence of results can be most efficiently translated into practice and be utilized in evidence-based clinical decision-making, viz. translational effectiveness.

### Translational effectiveness

As noted, translational effectiveness represents the translation of the best available evidence in the clinical practice to ensure its utilization in clinical decisions. Translational effectiveness fosters evidence-based revisions of clinical practice guidelines. It also encourages effectiveness-focused, patient-centered and evidence-based clinical decision-making. Translational effectiveness rests not only on the expertise of the clinical staff and the empowerment of patients, caregivers and stakeholders, but also, and most importantly on the best available evidence [8].

The pursuit of the best available evidence is the foundation of translational effectiveness and more generally of translational medicine in evidence-based health care. The best available evidence is obtained through a systematic process driven by a research question/hypothesis that is articulated about clearly stated criteria that pertain to the patient (P), the interventions (I) under consideration (C), for the sought clinical outcome (O), within a given timeline (T) and clinical setting (S). PICOTS is tested on the appropriate bibliometric sample, with tools of measurements designed to establish the level (e.g., CONSORT) and the quality of the evidence. Statistical and meta-analytical inferences, often enhanced by analyses of clinical relevance [9], converge into the formulation of the consensus of the best available evidence. Its dissemination to all stakeholders is key to increase their health literacy in order to ensure their full participation in the utilization of the best available evidence in clinical decisions, viz., translational effectiveness.

To be clear, translational effectiveness – and, in the perspective discussed above, translational health care – is anchored on obtaining the best available evidence, which emerges from highest quality research. High quality of research is obtained when errors are minimized.

In an early conceptualization [10], errors in research were presented as those situations that threaten the internal and the external validity of a research study – that is, conditions that impede either the study’s reproducibility, or its generalization. In point of fact, threats to internal and external validity [10] represent specific aspects of systematic errors (i.e., bias) in the research design, methodology and data analysis. Thence emerged a branch of science that seeks to understand, control and reduce risk of bias in research.

### Risk of bias and the best available evidence

It follows that the best available evidence comes from research with the fewest threats to internal and to external validity – that is to say, the fewest systematic errors: the lowest risk of bias. Quality of research, as defined in the field of research synthesis [11], has become synonymous with low bias and contained risk of bias [12-15].

Several years ago, the Cochrane group embarked on a new strategy for assessing the quality of research studies by examining potential sources of bias. Certain original areas of potential bias in research were identified, which pertain to (a) the sampling and the sample allocation process, to measurement, and to other related sources of errors (reliability of testing), (b) design issues, including blinding, selection and drop-out, and design-specific caveats, and (c) analysis-related biases.

A Risk of Bias tool was created (Cochrane Risk of Bias), which covered six specific domains:

1. selection bias,

2. performance bias,

3. detection bias,

4. attrition bias,

5. reporting bias, and

6. other research protocol-related biases.

Assessments were made within each domain by one or more items specific for certain aspects of the domain. Each items was scored in two distinct steps:

1. the support for judgment was intended to provide a succinct free-text description of the domain being queried;

2. each item was scored high, low, or unclear risk of material bias (defined here as “…bias of sufficient magnitude to have a notable effect on the results or conclusions…” [16]).

It was advocated that assessments across items in the tool should be critically summarized for each outcome within each report. These critical summaries were to inform the investigator so that the primary meta-analysis could be performed either only on studies at low risk of bias, or for the studies stratified according to risk of bias [16]. This is a form of acceptable sampling analysis designed to yield increased homogeneity of meta-analytical outcomes [17]. Alternatively, the homogeneity of the meta-analysis can be further enhanced by means of the more direct quality-effects meta-analysis inferential model [18].

Clearly, one among the major drawbacks of the Cochrane Risk of Bias tool is the subjective nature of its assessment protocol. In an effort to correct for this inherent weakness of the instrument, the Cochrane group produced detailed criteria for making judgments about the risk of bias from each individual item [16]. Moreover, Cochrane recommended that judgments be made independently by at least two people, with any discrepancies resolved by discussion [16]. This approach to increase the reliability of measurement in research synthesis protocols is akin to that described by us [19,20] and by AHRQ [21].

In an effort to aid clinicians and patients in making effective health care related decisions, AHRQ developed an alternative Risk of Bias instrument for enabling systematical evaluation of evidence reporting [22]. The AHRQ Risk of Bias instrument was created to monitor four primary domains:

1. risk of bias: design, methodology, analysis scoring – low, medium, high

2. consistency: extent of similarity in effect sizes across studies within a bibliome scoring – consistent, inconsistent, unknown

3. directness: unidirectional link between the interventions of interest and the sought outcome, as opposed to multiple links in a casual chain scoring – direct, indirect

4. precision: extent of certainty for estimate of effect with respect to the outcome scoring – precise, imprecise In addition, four secondary domains were identified:

a. Dose response association: pattern of a larger effect with greater exposure (Present/Not Present/Not Applicable or Not Tested)

a. Confounders: consideration of confounding variables (Present/Absent)

a. Strength of association: likelihood that the observed effect is large enough that it cannot have occurred solely as a result of bias from potential confounding factors (Strong/Weak)

a. Publication bias

The AHRQ Risk of Bias instrument is also designed to yield an overall grade of the estimated risk of bias in quality reporting:

•Strength of Evidence Grades (scored as high – moderate - low – insufficient)

This global assessment, in addition to incorporating the assessments above, also rates:

–major benefit

–major harm

–jointly benefits and harms

–outcomes most relevant to patients, clinicians, and stakeholders

The AHRQ Risk of Bias instrument suffers from the same two major limitations as the Cochrane tool:

1. lack of formal psychometric validation as most other tools in the field [21], and

2. providing a subjective and not quantifiable assessment.

To begin the process of engaging in a systematic dialectic of the two instruments in terms of their respective construct and content validity, it is necessary to validate each for reliability and validity either by means of the classic psychometric theory or generalizability (G) theory, which allows the simultaneous estimation of multiple sources of measurement error variance (i.e., facets) while generalizing the main findings across the different study facets. G theory is particularly useful in clinical care analysis of this type, because it permits the assessment of the reliability of clinical assessment pro-tocols. The reliability and minimal detectable changes across varied combinations of these facets are then simply calculated [23]. However, it is recommended that G theory determination follow classic theory psychometric assessment.

**Figure 2:**
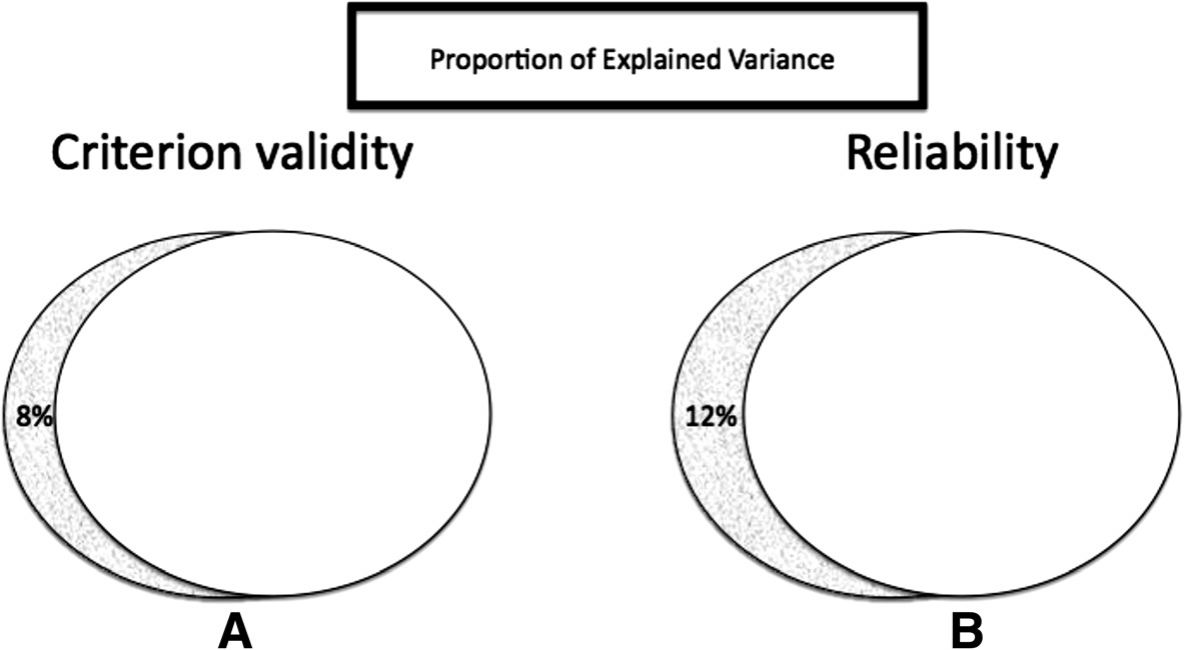
**Proportion of shared variance in criterion validity (A) and inter-rater reliability (B) in the AHRQ Risk of Bias instrument revised as described.** Two raters were trained and standardized [20] with the revised AHRQ Risk of Bias and with the R-Wong instrument, which has been previously validated [24]. Each rater independently produced ratings on a sample of research reports with both instruments on two separate occasions, 1–2 months apart. Pearson correlation coefficient was used to compute the respective associations. The figure shows Venn diagrams to illustrate the intersection between each two sets data used in the correlations. The overlap between the sets in each panel represents the proportion of shared variance for that correlation. The percent of unexplained variance is given in the insert of each panel.

Therefore, we have commenced a process of revision the AHRQ Risk of Bias instrument by rendering questions in primary domains quantifiable (scaled 1–4), which established the intra-rater reliability (r = 0.94, p < 0.05), and the criterion validity (r = 0.96, p < 0.05) for this instrument (Figure 2).

A similar revision of the Cochrane Risk of Bias tool may also yield promising validation data. G theory validation of both tools will follow. Together, these results will enable a critical and systematic dialectical comparison of the Cochrane and the AHRQ Risk of Bias measures.

## Discussion

The critical evaluation of the best available evidence is critical to patient-centered care, because biased research findings are fundamentally invalid and potentially harmful to the patient. Depending upon the tool of measurement, the validity of an instrument in a study is obtained by means of criterion validity through correlation coefficients. Criterion validity refers to the extent to which one measures or predicts the value of another measure or quality based on a previously well-established criterion. There are other domains of validity such as: construct validity and content validity that are rather more descriptive than quantitative. Reliability however is used to describe the consistency of a measure, the extent to which a measurement is repeatable. It is commonly assessed quantitatively by correlation coefficients. Inter-rater reliability is rendered as a Pearson correlation coefficient between two independent readers, and establishes equivalence of ratings produced by independent observers or readers. Intra-rater reliability is determined by repeated measurement performed by the same subject (rater/reader) at two different points in time to assess the correlation or strength of association of the two sets of scores.

To establish the reliability of research quality assessment tools it is necessary, as we previously noted [20]:

•a) to train multiple readers in sharing a common view for the cognitive interpretation of each item. Readers must possess declarative knowledge a factual form of information known to be static in nature a certain depth of knowledge and understanding of the facts about which they are reviewing the literature. They must also have procedural knowledge known as imperative knowledge that can be directly applied to a task in this case a clear understanding of the fundamental concepts of research methodology, design, analysis and inference.

•b) to train the readers to read and evaluate the quality of a set of papers independently and blindly. They must also be trained to self-monitor and self-assess their skills for the purpose of insuring quality control.

•c) to refine the process until the inter-rater correlation coefficient and Cohen coefficient of agreement are about 0.9 (over 81% shared variance). This will establishes that the degree of attained agreement among well-trained readers is beyond chance.

•d) to obtain independent and blind reading assessments from readers on reports under study.

•e) to compute means and standard deviation of scores for each question across the reports, repeat process if the coefficient of variations are greater than 5% (i.e., less than 5% error among the readers across each questions).

The quantification provided by instruments validated in such a manner to assess the quality and the relative lack of bias in the research evidence allows for the analysis of the scores by means of the acceptable sampling protocol. Acceptance sampling is a statistical procedure that uses statistical sampling to determine whether a given lot, in this case evidence gathered from an identified set of published reports, should be accepted or rejected [12,25]. Acceptable sampling of the best available evidence can be obtained by:

•convention: accept the top 10 percentile of papers based on the score of the quality of the evidence (e.g., low Risk of Bias);

•confidence interval (CI^95^): accept the papers whose scores fall at of beyond the upper confidence limit at 95%, obtained with mean and variance of the scores of the entire bibliome;

•statistical analysis: accept the papers that sustain sequential repeated Friedman analysis.

To be clear, the Friedman test is a non-parametric equivalent of the analysis of variance for factorial designs. The process requires the 4-E process outlined below:

•establishing a significant Friedman outcome, which indicates significant differences in scores among the individual reports being tested for quality;

•examining marginal means and standard deviations to identify inconsistencies, and to identify the uniformly strong reports across all the domains tested by the quality instrument

•excluding those reports that show quality weakness or bias

•executing the Friedman analysis again, and repeating the 4-E process as many times as necessary, in a statistical process akin to hierarchical regression, to eliminate the evidence reports that exhibit egregious weakness, based on the analysis of the marginal values, and to retain only the group of report that harbor homogeneously strong evidence.

Taken together, and considering the domain and the structure of both tools, expectations are that these analyses will confirm that these instruments are two related entities, each measuring distinct aspects of bias. We anticipate that future research will establish that both tools assess complementary sub-constructs of one and the same archetype meta-construct of research quality.

## Competing interests

The authors declare that they have no competing interests.

## Authors’ contributions

This paper was planned by Dr. FC, and he is responsible for the initial drafting of the manuscript. AB provided the overall conception of the project, and crafting of the fundamental dialectics between the Cochrane and the AHRQ Risk of Bias instruments. Dr. JM did the initial exploratory work on the AHRQ Risk of Bias instrument, which was then substantially advanced and systematically analyzed by PP with the assistance of VL, LG, MoD and Dr. MaD. All authors contributed critical revisions of the manuscript for important intellectual content, and read and approved the final manuscript.
